# Classification of triple negative breast cancer by epithelial mesenchymal transition and the tumor immune microenvironment

**DOI:** 10.1038/s41598-022-13428-2

**Published:** 2022-06-10

**Authors:** Francesc Font-Clos, Stefano Zapperi, Caterina A. M. La Porta

**Affiliations:** 1 Center for Complexity and Biosystems, University of Milan, via Celoria 16, 20133 Milano, Italy; 2 Department of Physics, University of Milan, Via Celoria 16, 20133 Milano, Italy; 3 CNR - Consiglio Nazionale delle Ricerche, Istituto di Chimica della Materia Condensata e di Tecnologie per l’Energia, Via R. Cozzi 53, 20125 Milano, Italy; 4 Department of Environmental Science and Policy, University of Milan, via Celoria 26, 20133 Milano, Italy; 5 CNR - Consiglio Nazionale delle Ricerche, Istituto di Biofisica, via Via De Marini 6, 16149 Genova, Italy

**Keywords:** Gene regulatory networks, Breast cancer

## Abstract

Triple-negative breast cancer (TNBC) accounts for about 15–20% of all breast cancers and differs from other invasive breast cancer types because it grows and spreads rapidly, it has limited treatment options and typically worse prognosis. Since TNBC does not express estrogen or progesterone receptors and little or no human epidermal growth factor receptor (HER2) proteins are present, hormone therapy and drugs targeting HER2 are not helpful, leaving chemotherapy only as the main systemic treatment option. In this context, it would be important to find molecular signatures able to stratify patients into high and low risk groups. This would allow oncologists to suggest the best therapeutic strategy in a personalized way, avoiding unnecessary toxicity and reducing the high costs of treatment. Here we compare two independent patient stratification strategies for TNBC based on gene expression data: The first is focusing on the epithelial mesenchymal transition (EMT) and the second on the tumor immune microenvironment. Our results show that the two stratification strategies are not directly related, suggesting that the aggressiveness of the tumor can be due to a multitude of unrelated factors. In particular, the EMT stratification is able to identify a high-risk population with high immune markers that is, however, not properly classified by the tumor immune microenvironment based strategy.

## Introduction

Breast cancer accounts for 25% of all newly diagnosed cancer cases in women around the world. Despite clinical improvements introduced in the past decades, predicting the clinical outcome of individual patients is still an open challenge^1^. This is a very important goal since current treatments are costly and have important side effects, which are detrimental for the patients quality of life. Hence, being able to predict which patients will be most likely to benefit from a given treatment would help establish personalized therapies and avoid overtreatment. The difficulty of this challenge stems from to the considerable heterogeneity of breast cancer, even within the standard molecular subtypes in which this tumor is usually classified^2,3^. Breast cancer subtypes are based on the expression level of estrogen receptor (ER), progesteron receptor (PR), the human epidermal growth factor receptor 2 (HER2) and the proliferation marker Ki67. In particular, the four subtypes that are mostly used to classify breast cancer are Luminal A (ER and/or PR+, HER2−, Ki67 low), Luminal B (ER and/or PR+, HER2−, Ki67 high), HER2 positive (HER2+) and triple negative (ER−, PR−, HER2−)^2–5^.

Stratification of breast cancer patients within each of the four subtypes has been attempted using a plethora of gene expression tests based on the expression level of gene panels either empirically selected^6,7^ or resulting from machine learning classification of whole transcriptomic data^8,9^. Older tests have mostly been applied to to the Luminal A breast cancer subtype and essentially measure the proliferation level. Machine learning based methods have been shown to suffer from overfitting^10,11^. The problem arises when a machine learning algorithm tries to classify a high-dimensional object by using a small training set^11^. When the dimension of the object (around 20000 genes in the case of the human transcriptome) is larger than the number of samples in the training set, the predictive power of the resulting classifier is poor.

Stratifying patients with triple negative breast cancer (TNBC) is an issue that has been addressed only in recent years^12–14^. This breast cancer subtype is the most aggressive of the four, showing poor prognosis, particularly when metastasis are present, and is highly heterogeneous^15^. The classification scheme proposed by Lehmann et al.^12^ and later refined by the same group^16^ is based on the clustering of gene expression data, leading to six subtypes which display differential response to treatment^12^, but no statistically significant differences in relapse-free survival^16^.

In a recent work, we introduced and validated ARIADNE, a general algorithmic strategy to assess the risk of metastasis of patients with TNBC based on the identification of hybrid epithelial/mesenchymal phenotypes from gene expression data^17^. The method is based on a Boolean network model that is able to efficiently classify cell phenotypes by mapping gene expression data into a complex landscape whose topographic features represent important biological aspects of the cells^18^. The epithelial–mesenchymal transition (EMT) describes how polarized epithelial (E) cells transform into mesenchymal (M) cells by losing cell polarity and down-regulating adhesion molecules, such as E-cadherin. M cells tend to be more motile, suggesting that EMT could be associated with metastatic capabilities^19–22^. Recent work shows that the EMT can also involve hybrid E/M states^23^ where cells display a mix of markers, characteristic of E and M cells^24–26^. These hybrid states combine invasive capabilities and intracellular adhesion^27,28^ and are associated to extremely aggressive tumors^23,29–31^. Several EMT scores have been proposed to determine the E or M character of a tumor sample based on gene expression data^32^. We showed that ARIADNE correlates with other EMT scores but it is more specific in identifying hybrid phenotypes, which is essential to stratify patients^17^.

Due to the possible involvement of the immune system in modulating the phenotype of tumor cells, a recent paper suggested that immunological metasignatures could stratify TNBC patients^33,34^. In particular, the authors focus on the tumor immune microenvironment, considering gene expression profiles of matched tumor, epithelial and stromal compartments from TBNC patients^33^. Using these data, the authors classify patients according to specific combinations of gene expression metasignatures that are able to stratify patients clinical outcomes^33^. The paper also shows that each of these immunological subtypes expresses distinct patterns of immune related gene markers (i.e. immune suppression, IL-17 induction and production, cell death, neutrophils, type I Interpherons (IFN), cytotoxic activity and antigen presentation).

Given that previous papers show that the same TNBC patients can effectively be stratified by two independent strategies, one based on the EMT^17^ and the other based on the tumor immune microenvironment^33^, we decided to investigate whether the two strategies are related. In other words, do patients considered at high/low risk according to the EMT based approach also show distinct immunological signatures? To address this question, we use ARIADNE to analyze gene expression data from patients included in the study of the TNBC tumor immune microenvironment^33^ and then check if the groups selected by ARIADNE show any peculiar differences in the expression of immune-related genes.

## Methods

### Matching different datasets

Gene expression data analyzed in Gruosso et al.^33^ are accessible in the GEO database under accession numbers GSE88715 (for gene expression from stromal and epithelial compartments) and GSE88847 (for bulk tumor gene expression). Survival data can be obtained from an earlier dataset (GSE58644) which contains gene expression data for the same patients together with others^35^. We could not find an indication of how gene expression data from GSE88847 can be matched with the survival data in GSE58644. To solve this problem, we compute the correlation of pairs of transcriptomes from GSE88847 and GSE58644. We find that for each sample in GSE88847 there is a matching sample in GSE58644 that has a much larger correlation coefficient than the rest. We then verify the potential matching with clinical data (i.e. tumor size and age), and exclude four cases where the matching is not reliable because clinical data do not agree.

### Data normalization

**Figure 1:**
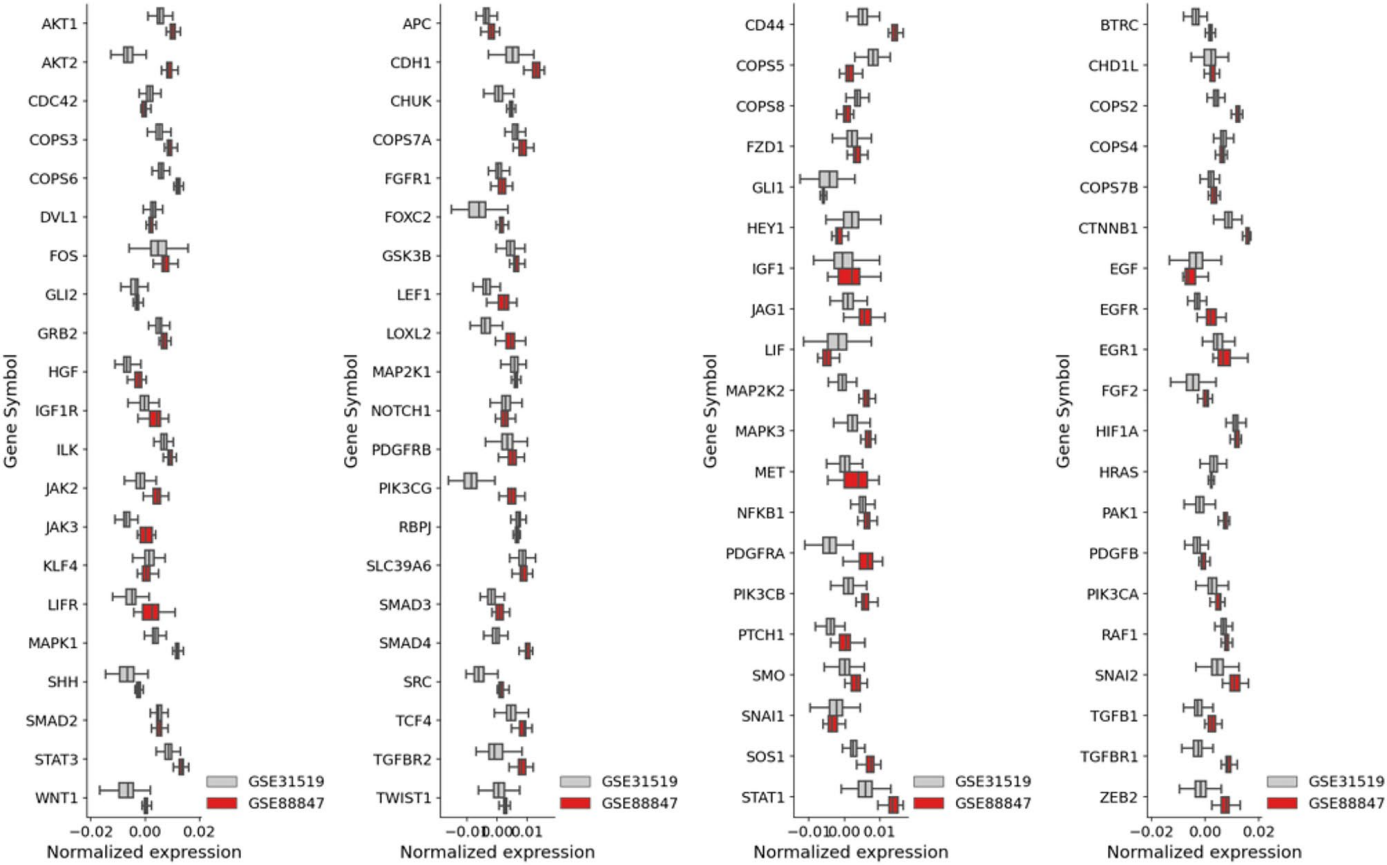
Data normalization is consistent across datasets. Boxplot of normalized expression for genes used as part of the ARIADNE score computation, comparing the dataset used to train ARIADNE (GSE31519) in^17^ and the dataset analysed in this manuscript (GSE88847).

We normalize data from GSE88847 and GSE88715 by following the procedure adopted by Karn et al.^36^ for GSE31519. To be precise:

1. log2 transformation of MAS5 values
2. median centering of arrays
3. magnitude normalization of arrays.

where magnitude normalization must be understood as setting the sum of squares of all samples to one. This is because ARIADNE was trained on GSE31519^17^, and in this manuscript we do not re-train ARIADNE, but rather tackle the challenge of reusing the parameters obtained in Font-Clos et al.^17^ to compute the score of a new dataset. Therefore, it is crucial to use the same normalization as in the training data. When comparing different datasets one should also keep in mind that additional sources of variability could come from differences in sample preparation across different studies. Figure 1 shows the distribution of normalized expression for the genes used in the score computation, comparing the training data of ARIADNE (i.e. GSE31519) with the data newly analyzed in the present paper (i.e. GSE88847 and GSE88715). We then compute the ARIADNE score as explained in Font-Clos et al.^17^ for the samples in GSE88847 and GSE88715. After computing the raw ARIADNE score, which is an integer value, we define the high and low groups simply by sorting and splitting the dataset into two groups, high and low.

**Figure 2:**
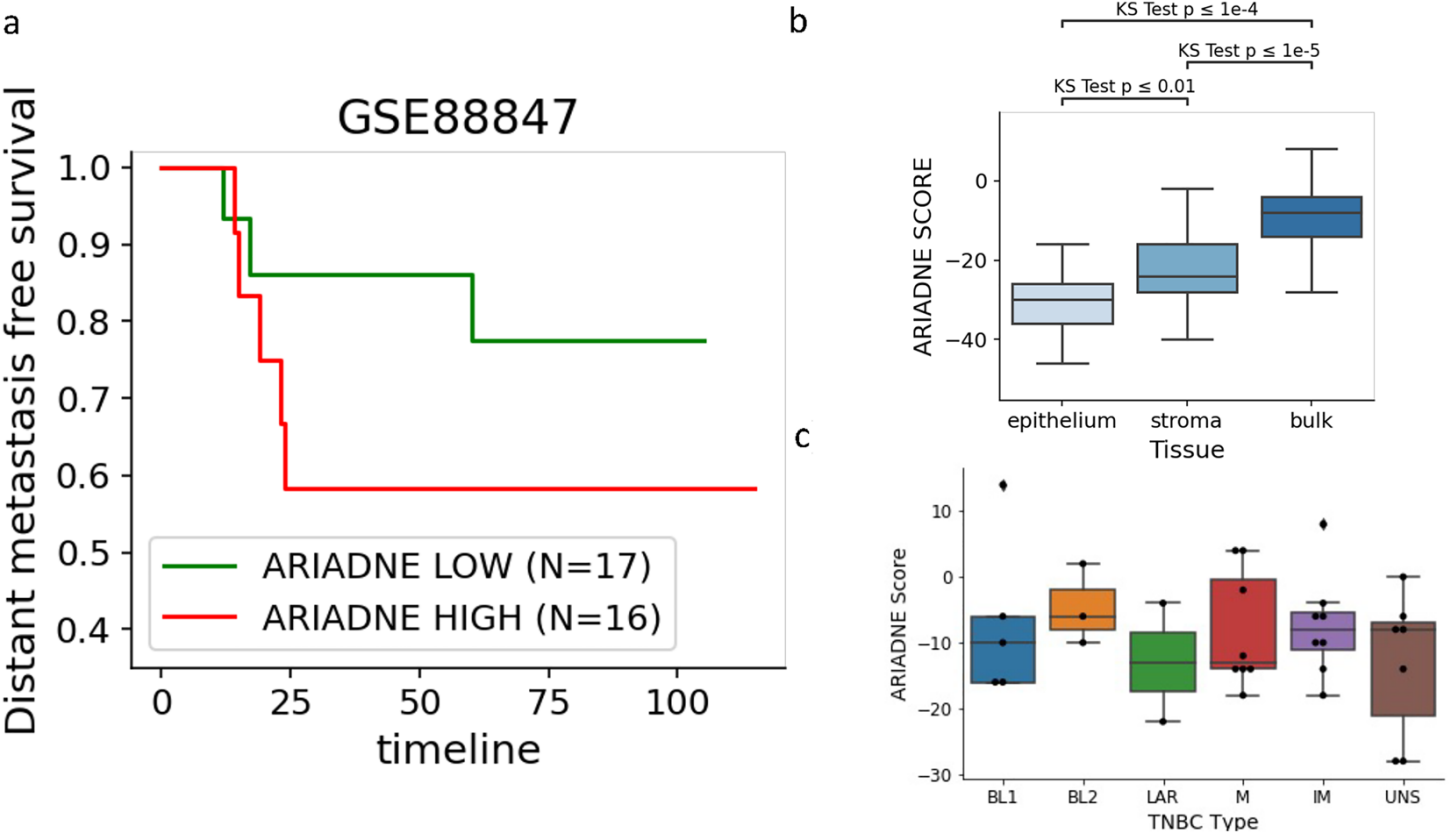
ARIADNE score predicts survival. (**a**) Survival curves for low (green) and high (red) patients as stratified by the ARIADNE score. The panel shows that ARIADNE is able to stratify triple-negative breast cancer patients on unseen data without the need to retrain the algorithm. (**b**) ARIADNE score for gene expression data from the epithelium and stroma adjacent tissues, compared to those from the main bulk tissue. A higher value of ARIADNE is associated to more hybrid and aggressive phenotypes. (**c**) ARIADNE scores associated with different TNBC subtypes according to^12^.

**Figure 3:**
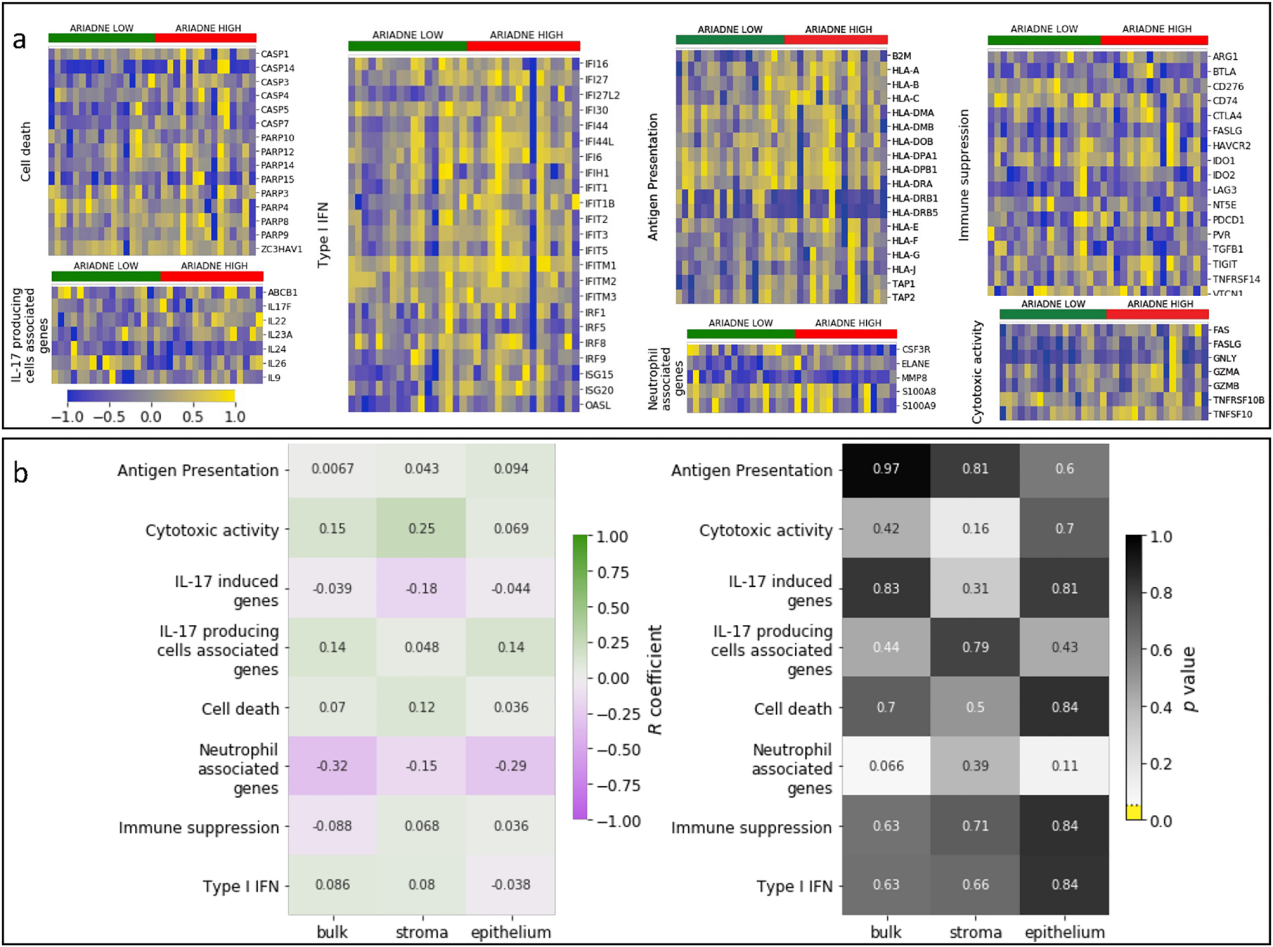
ARIADNE score is not correlated with immunological gene sets. The heatmaps report the normalized gene expression of the bulk cancer samples for the immunological gene sets studied in^33^. (**a**) The heatmap shows normalized gene expression values sorted according to the value of the ARIADNE score. (**b**) Cross correlation analysis between mean gene expression value in each pathway and the ARIADNE score leads to small correlation coefficient. No pathway yields significant correlations ($p<0.05$) in bulk, stroma or epithelium. Heatmaps are done in python (version 3.7.4) using the seaborn package (version 0.11.2) (https://seaborn.pydata.org).

### Calculation of pathway deregulation scores

Pathway Deregulation Scores (PDS) were first introduced by Drier et al.^37^ as a way of quantifying the overall deregulation of a given pathway with respect to a reference sample by fitting a non-parametric, non-linear one-dimensional curve through the “middle” of the transcriptomic data, in the subspace generated by the genes of that pathway. In practice, this is usually done via the *principal curve* algorithm^38^, although other procedures would be acceptable. We follow the steps of Drier et al.^37^, except for the following modification that we introduced in a previous paper^39^. We place the value of 0 the mean value of the reference sample, instead of at the extremal point of the curve. This modification alters the resulting PDS only by a linear shift, but makes the results more robust against the variability of the reference samples, as discussed in Font-Clos et.^39^. We compute PDS for the immunological gene sets reported in Gruosso et al.^33^ and for a subset of immunologically related “hallmark gene sets” obtained from msigdb^40^. Boxplots show the distribution of PDS values, for each pathway, both for “ARIADNE low” samples (green) and for “ARIADNE high” samples (red).

### Tumor immune microenvironment metasignatures

The list of genes corresponding to the metasignatures proposed by Gruosso et al.^33^ are obtained from https://github.com/bhklab/EpiStromaImmune/. We focus our analysis on the “Immune” (CDSig1), “Fibrosis (CDSig3), “Cholesterol” (EDSig2) and “Interferon (IFN)” (EDSig5) metasignatures and use them to classify patients into groups following the algorithm described in Gruosso et al.^33^ and reported in https://github.com/bhklab/EpiStromaImmune/. In particular, we first construct two groups—“Immune high/Fibrosis low” and “Immune low/Fibrosis high”—representing 60% and 40% of the samples respectively. We define samples that end up in both groups as “Intermediate”. This differs slightly from Gruosso et al.^33^ where those samples are later re-assigned to one of the two classes. We then refine the classification for the samples in the “Immune high/Fibrosis low” group by constructing two additional groups based on the “Cholesterol” and “Interferon” metasignatures, each containing 50% of the samples^33^. We compute the metasignatures for GSE88847 and GSE31519. When comparing the metasignature with ARIADNE, we consider two groups for GSE88847 (high and low) and three groups for GSE31519 (low, med and high, as in Font-Clos et al.^17^) owing to the larger sample size of the second dataset.

### TNBC subtypes

We establish the subtype (TNBCtype) of the samples in GSE88847 according to Lehmann et al.^12^ submitting the GSE88847 gene expression dataset to the TNBCtype server (https://cbc.app.vumc.org/tnbc/).

### Computation of survival curves

We use the lifelines python package to compute survival curves in Fig. 2a using the Kaplan-Meyer approach.

**Figure 4:**
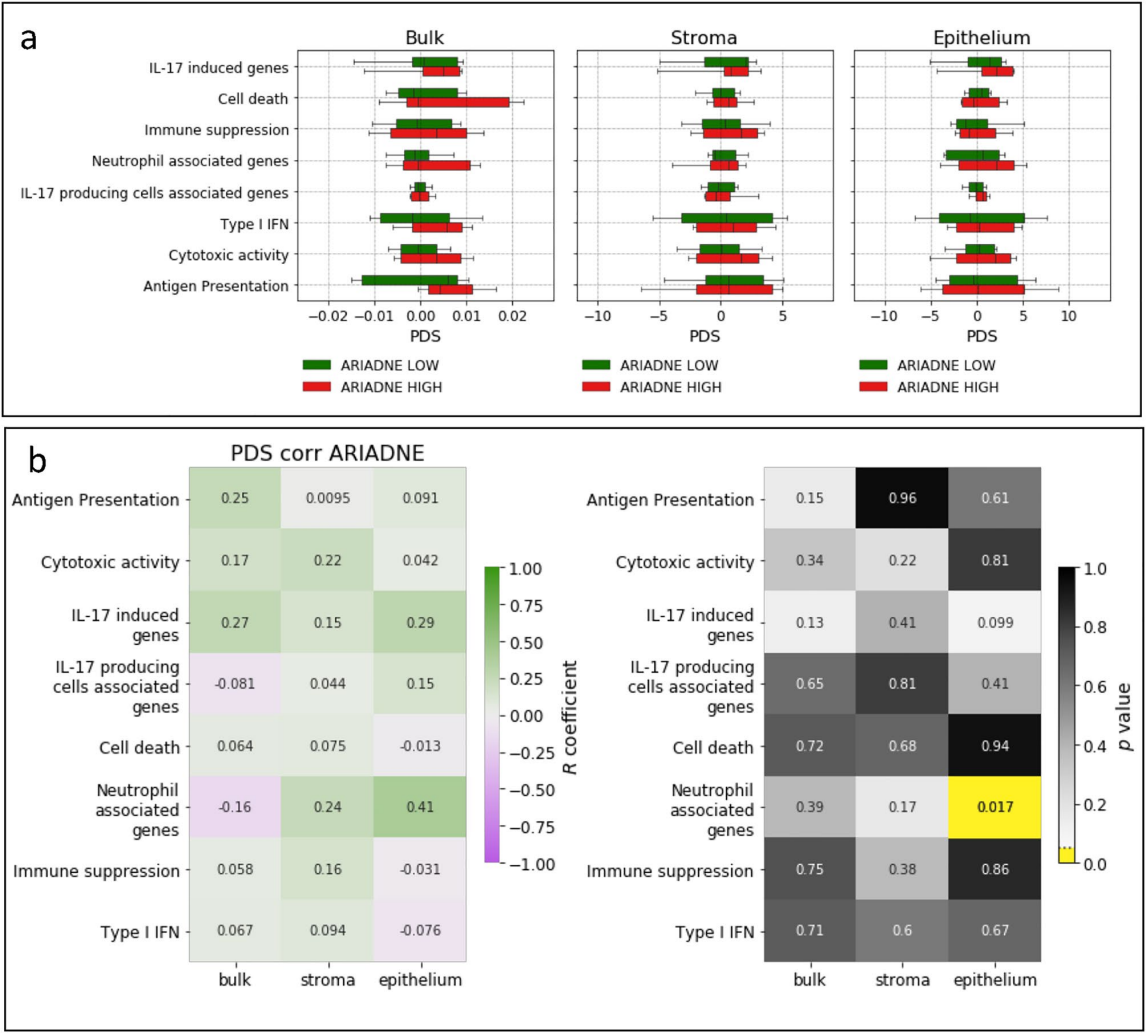
ARIADNE score is not correlated with pathway deregulation score of immunological gene sets. (**a**) Boxplots of the PDS of each immunological gene set separated by ARIADNE class for bulk tumor, stroma and epithelium. (**b**) Cross-correlation coefficients and *p*-values for the correlation between ARIADNE score and PDS.

**Figure 5:**
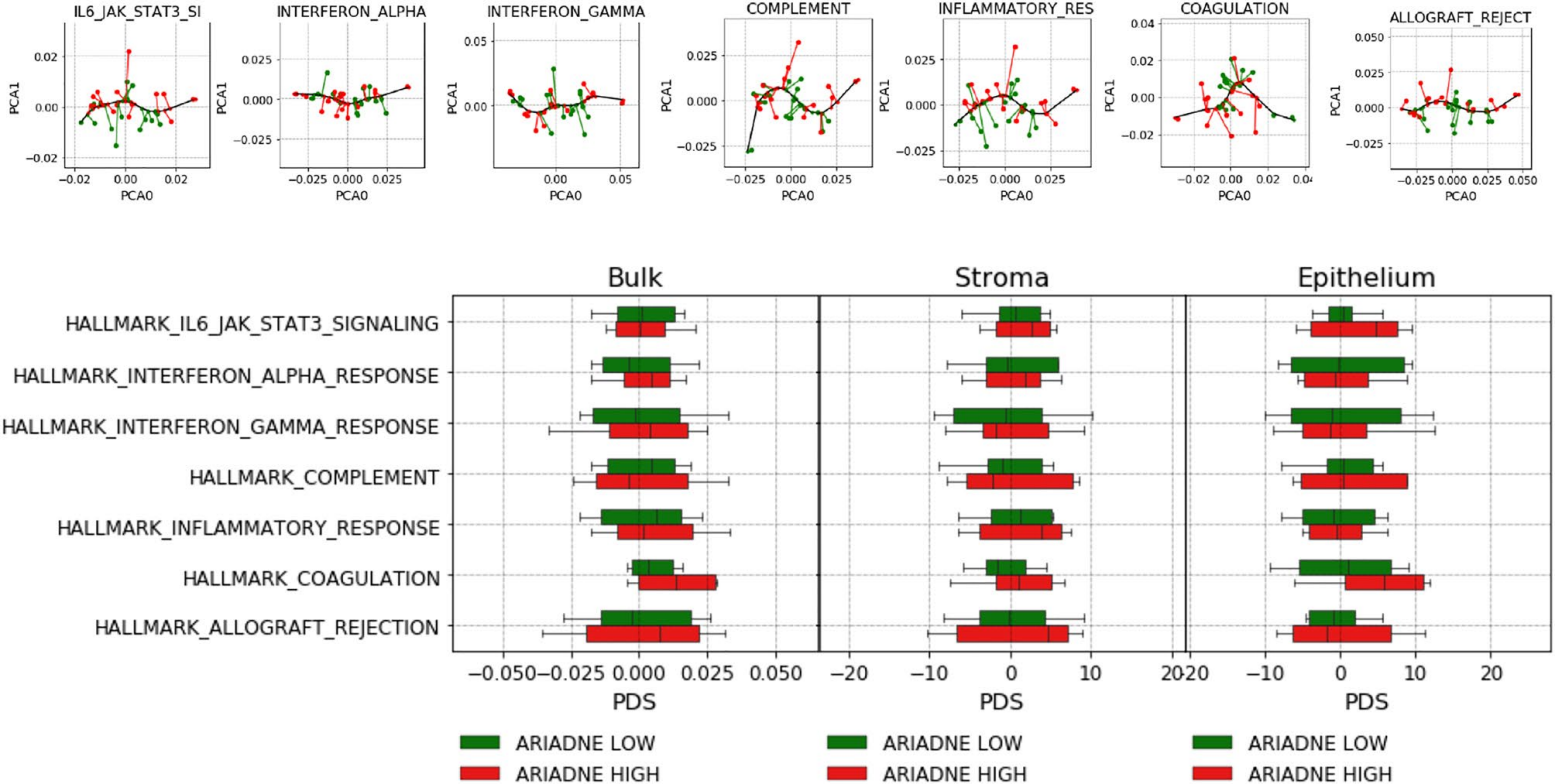
ARIADNE score is not correlated with pathway deregulation score for immune related hallmark pathways. Projections of bulk tumor samples of a set of immune related hallmark pathways (top panels). Boxplots of the PDS of each pathway separated by ARIADNE class for bulk tumor, stroma and epithelium (bottom panels).

**Figure 6:**
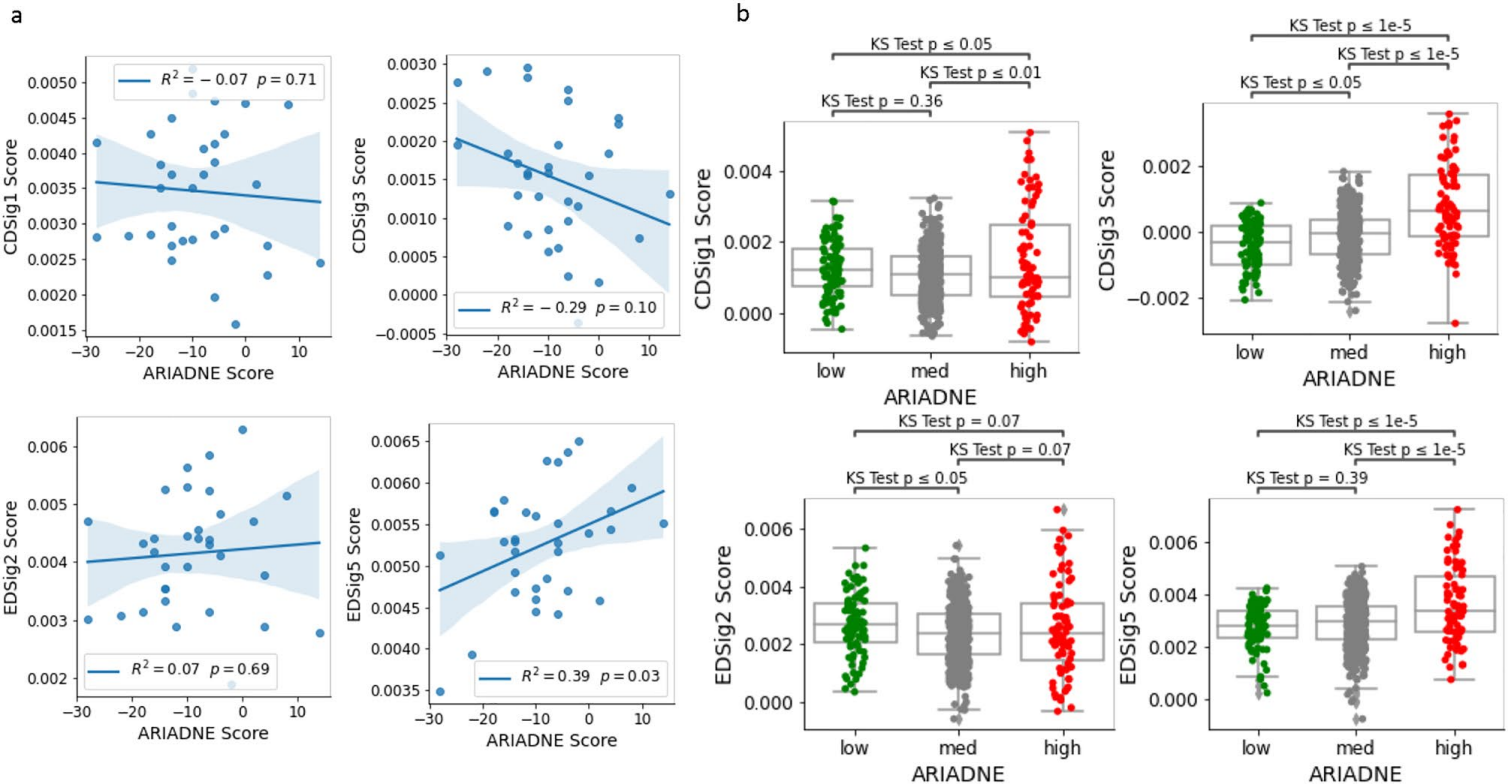
ARIADNE is associated with immune metasignatures in a large dataset. (**a**) Cross-correlation between immune metasignature scores and ARIADNE score for samples in the GSE88847 dataset. (**b**) Metasignature scores for groups classified according to the ARIADNE score for the GSE31519 dataset. Statistical significance is established using the KS test.

**Figure 7:**
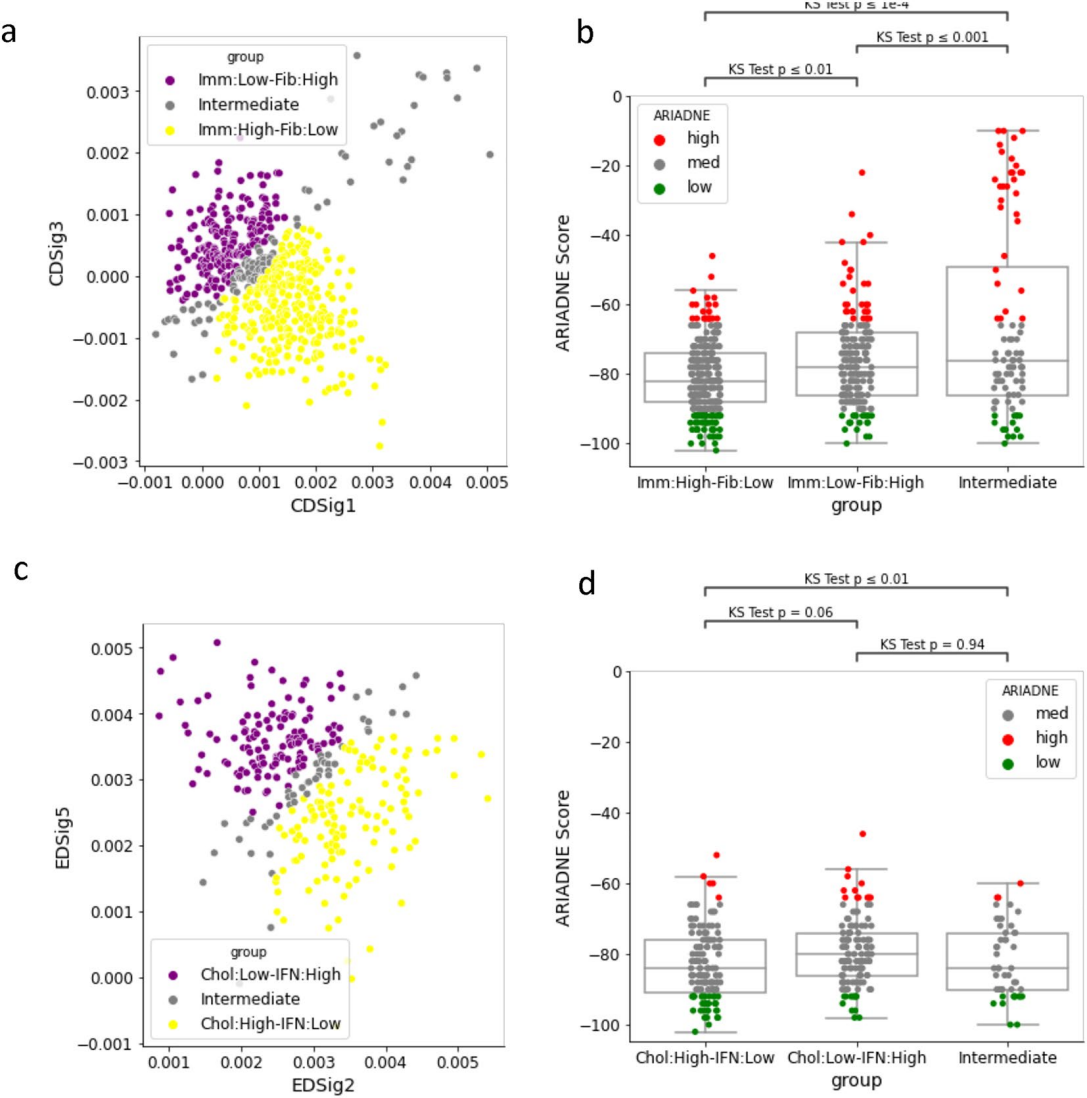
Groups based on immune metasignatures and ARIADNE scores. (**a**) Classification of samples in the GSE31519 dataset into “Immune high”/“Fibrosis low” and “Immune low”/“Fibrosis high” groups. (**b**) The ARIADNE score computed for samples in the GSE31519 dataset divided according to the “Immune high”/“Fibrosis low” and “Immune low”/“Fibrosis high” classification^33^. (**c**) Classification of samples in the GSE31519 dataset into “Cholesterol low”/“Interferon high” and “Cholesterol high”/“Interferon low” groups. (**d**) The ARIADNE score computed for samples in the “Immune high”/“Fibrosis low” divided according to the “Cholesterol low”/“Interferon high” and “Cholesterol high”/“Interferon low” classification^33^. Statistical significance is established using the KS test.

### Statistical analysis

In correlation plots, statistical significance is established through linear regression. Statistical differences in the distributions of ARIADNE scores for immune-related groups and among tissues are established using the Kolmogorov-Smirnov (KS) test.

### Statement

All methods were carried out in accordance with relevant guidelines and regulations.

## Results

We access gene expression data from TNBC patients taken from the tumor (GSE88847) and from adjacent tissues (stroma and epithelium) already analyzed in Gruosso et al.^33^ and match them to survival data^35^ as described in the Methods section. We then stratify the patients according to the score provided by the ARIADNE algorithm^17^ which maps gene expression data into the states of a Boolean network model simulating gene regulatory interactions responsible for the EMT^18^. The algorithm was already trained and cross-validated on a large cohort of TNBC patients (GSE31519^41^) and was able to identify low and high risk patients based on the presence of hybrid E/M characteristics^17^. As shown in Fig. 2a, ARIADNE successfully stratifies patients in two risk classes, a low risk class with high survival and a high risk class with lower survival. We then applied ARIADNE also to gene expression data measured in tissues adjacent to the bulk tumor (i.e. stroma and epithelium). As shown in Fig. 2b, the ARIADNE score, which measures the presence of hybrid E/M cells, is larger in the tumor bulk and smaller in the epithelium with intermediate scores found in the stroma. The differences are statistically significant as demonstrated by the KS test. This suggests an increasing presence of hybrid E/M phenotypes from the epithelium to the stroma and finally to the bulk tumor. We also establish the TNBC subtype of the tumor samples according to Lehmann et al.^12^. As shown in Fig. 2c, samples are scattered across the six subtypes independently of their ARIADNE score.

Having confirmed that this cohort of patients can be effectively stratified by ARIADNE based on the EMT status of the tumor, we consider signatures related to the tumor immune microenvironment. To this end, we first consider the immunological gene sets considered in Gruosso et al.^33^ and analyze if their expression correlates with the score produced by ARIADNE. As shown in Fig. 3a, we can not see any clear pattern in the gene expression values measured from bulk tumor samples when those are sorted according to their ARIADNE scores. To be more quantitative, we compute the cross-correlation between the ARIADNE score and the mean expression value within each gene set. The results displayed in Fig. 3b show that correlation coefficients are rather small and not statistically significant, even when the significance level is not particularly strict (i.e. $\alpha =0.05$ without multiple testing correction). These negative results hold for all sample types: Bulk tumor, stroma and epithelium.

To obtain a more precise assessment of the possible relation between the stratification obtained by ARIADNE and the tumor immune microenvironment, we compute pathway deregulation scores (PDS)^37^. The method quantifies the overall deregulation of a given pathway with respect to a reference sample, by fitting a non-parametric non-linear one-dimensional curve through the gene expression data relative to each pathway (see Methods for details). We apply the method using again the same gene sets (Fig. 4a) and then compute a cross-correlation between PDS and ARIADNE score. Again correlations are weak and not statistically significant (Fig. 4)b. We also repeat the same analysis for a set of immune related hallmark pathways^40^. As shown in Fig. 5, we do not detect any significant correlation between PDS and ARIADNE score.

Finally, we consider the immunological metasignatures defined in Gruosso et al.^33^ and compare their value with ARIADNE. In particular, we consider the “Immune” (CDSig2), “Fibrosis” (CDSig4), “Cholesterol” (EDSig2) and “Interferon” (EDsig5) metasignatures used in Gruosso et al.^33^ to stratify patients. Cross-correlation analysis for the data in GSE88847 does not reveal significant correlations between the scores, except in one case (see Fig. 6a). To check if the lack of correlation is due to the relatively small size of the dataset, we also consider a larger dataset (i.e. GSE31519). As shown in Fig. 6b, the group of patients with high ARIADNE score displays a small but statistically significant enrichment in all the metasignatures. We then proceed as in Gruosso et al.^33^ and define groups based on combinations of the metasignatures. In particular, we first divide patients in two classes: “Immune high”/“Fibrosis low” and “Immune low”/“Fibrosis high” (see Fig. 7a). As shown in Fig. 7b, there is a small but statistically significant difference in ARIADNE score between the two classes. Remarkably, the largest differences in ARIADNE score are observed in patients that fall in both groups and that we classify as “intermediate” (Fig. 7a). Our result is consistent with Fig. 6b showing that a number of patients with high ARIADNE score and also high immune and fibrosis markers. We also consider a sub-classification of the “Immune high”/“Fibrosis low” group into “Cholesterol low”/“Interferon high” and “Cholesterol high”/“Interferon low” groups (Fig. 7c), finding no significant association with ARIADNE score (Fig. 7d).

## Conclusions

The possibility to stratify TBNC patients is a crucial aspect to build personalized treatments, which would be particularly relevant for this breast cancer subtype where no specific therapeutic strategy is available. Several patients stratification strategies based on gene expression data have been proposed in the literature. The most widely used classification of TNBC was proposed by Lehmann et al.^12^ and it is based on automatic clustering of gene expression data and resulted in six subgroups, later refined into four^16^. The Lehmann classification showed promising results in identifying patients who respond to treatment^12^, but limited success in identifying relapse-free surviving patients^16^.

Alternative patient stratification strategies for TNBC are built on specific biological processes known to affect clinical outcome, rather than performing an unsupervised analysis of gene expression data as in the case of Lehmann et al.^12^. In this paper, we compared two of these strategies, one based on the EMT, which we introduced in a recent paper^17^, and the other based on the tumor immune microenvironment^33^. Our analysis suggests that our EMT based stratification successfully identifies high risk patients in a way that is largely independent of the tumor immune microenvironment and the Lehmann subtyping. Our analysis, however, reveals a small fraction of patients with high ARIADNE score and large metasignature scores that is not properly classified according the the categories proposed in Gruosso et al.^33^. This point is particularly interesting since it illustrates the potential of ARIADNE in identifying patients that fall into a grey area when classified with immune categories. Apart from this subpopulation, other patients classified as high or low risk by ARIADNE do not display a peculiar profile in terms of their tumor immune microenvironment.

## Data availability

The datasets analyzed during the current study are available in the GEO repository under accession numbers GSE88847 https://www.ncbi.nlm.nih.gov/geo/query/acc.cgi?acc=GSE88847, GSE88715 https://www.ncbi.nlm.nih.gov/geo/query/acc.cgi?acc=GSE88715, and https://www.ncbi.nlm.nih.gov/geo/query/acc.cgi?acc=GSE31519 GSE31519.
